# Immediate and long-term efficacy of laterally-wedged insoles on persons with bilateral medial knee osteoarthritis during walking

**DOI:** 10.1186/s12938-015-0040-6

**Published:** 2015-05-14

**Authors:** Wei-Chun Hsu, You-Cai Jhong, Hao-Ling Chen, Yi-Jia Lin, Li-Fei Chen, Lin-Fen Hsieh

**Affiliations:** Graduate Institute of Biomedical Engineering, National Taiwan University of Science and Technology, Taipei, Taiwan; National Defense Medical Center, Taipei, Taiwan; School of Occupational Therapy, National Taiwan University, Taipei, Taiwan; Department of Physical Medicine and Rehabilitation, Shin Kong Wu Ho-Su Memorial Hospital, Taipei, Taiwan; School of Medicine, Fu Jen Catholic University, New Taipei City, Taiwan

**Keywords:** Osteoarthritis, Knee, Laterally-wedged insoles, Joint kinematics, Joint kinetics, Gait adaptation

## Abstract

**Background:**

The current study aimed to investigate the immediate and long-term effects of laterally-wedged (LW) insoles on the knee loadings, the knee abductor moment (KAM) in particular, and the compensatory changes at other lower limb joints in patients with bilateral medial knee osteoarthritis during level walking with and without LW insoles.

**Methods:**

Older adults with bilateral medial knee OA (age 66 ± 5.3 years; height 156 ± 4.9 cm; mass 60 ± 5.1 kg; leg length 83.72 ± 3.64 cm) were studied using computerized gait analysis initially (Baseline) and 6 weeks after using LW insoles (Follow-up) during barefoot walking and walking with LW insoles (7° of lateral inclination, with medial arch support). The three-dimensional angles and internal moments at the lower limb joints, as well as the ground reaction forces, were obtained using a motion analysis system and two forceplates. Key features of all the variables were compared using paired *t* tests for immediate effects (barefoot vs. LW) and for long-term effects (Baseline vs. Follow-up). The symptomatic severity (WOMAC Index) was also evaluated (Baseline vs. Follow-up).

**Results:**

The KAM with LW insoles at Baseline was significantly reduced when compared to the barefoot condition (*p* < 0.05), suggesting that the LW insoles were effective in reducing unfavorable loadings at the knee immediately upon wearing the insoles. After 6 weeks of wearing LW insoles (Follow-up), no significant changes were found in most of the biomechanical variables, including KAM (*p* > 0.05), when compared to Baseline with LW insoles. However, a specific gait adaptation with reduced knee loading was revealed when walking without LW insoles, i.e., for the barefoot condition (*p* < 0.05).

**Conclusions:**

After long-term use of LW insoles, the pain and physical function were improved with decreased peak KAM. A specific gait adaptation with reduced KAM was also found when walking without LW insoles. These results indicate a positive long-term effect in persons with bilateral medial knee OA, both as an orthosis to assist walking, and as a treatment intervention to facilitate gait adaptations in favor of reduced KAM.

## Background

Knee osteoarthritis (OA) is a degenerative joint disease commonly seen in the middle-aged and older population. Patients with knee OA often suffer from pain, muscle weakness, swelling, stiffness, limited range of motion, as well as joint deformity and malalignment [1, 2], which may lead to abnormal gait patterns and joint loadings [2–4], as well as reduced quality of life [5]. Treatments such as analgesics and non-steroidal anti-inflammatory drugs are effective in relieving pain and inflammation but do not normally address the underlying biomechanical changes that are related to the causes and/or progression of the disease. Laterally-wedged (LW) insoles aim to reduce the unfavorable loadings at the knee by controlling the frontal alignment of the foot. However, previous studies on both immediate and long-term effects of LW insoles have produced mixed outcome in terms of the improvement in pain, physical function and quality of life [6–8]. Most of the previous studies on the long-term effects were based on subjective evaluations, such as WOMAC scores [6, 7, 9–11]. Quantitative biomechanical assessment of the long-term effects of LW insoles on gait performance has been limited [12–14].

Among the limited studies on the long-term effects of LW insoles, most focused on the assessment of the clinical outcome and static alignment [6, 7, 9–11], giving controversial results. Laterally wedged insoles were shown to be associated with early improvements in pain, stiffness and functional limitations [6, 7], as well as significant reductions in the varus alignment based on radiographic findings [11]. However, no significant symptomatic and/or structural improvement was found in patients with medial knee OA after wearing LW insoles long-term [9, 10]. It is noted that all the above-mentioned previous assessment studies were performed while wearing the insoles. It remains unclear whether the long-term use of LW would have the potential to achieve a therapeutic gait adaptation, such as gait with reduced harmful knee loadings. The presence of a gait adaptation after prolonged use of the LW insoles can be assessed only with the insoles removed, i.e., during barefoot walking.

Another factor that affects the effectiveness of LW insoles in reducing the unfavorable loadings at the knee is the compensatory changes at the ankle, which could potentially diminish the intended lateral shifting of the ground reaction force (GRF) vector when wearing the LW insoles. Previous studies have proposed LW insoles with arch supports or sub-talar strapping to reduce the attenuation effects of the ankle, but have produced controversial results [14–17]. Some authors showed that LW insoles with arch supports and sub-talar strapping helped reduce ankle eversion in young healthy subjects [8, 16, 17], while others reported otherwise [15]. Note that these results were immediate effects and may be very different from those after prolonged use of LW insoles.

Among the loading components, the knee abductor moment (KAM), i.e., moment provided by the knee abductors, has been shown to be associated with knee pain, severity of degenerated changes on the condyle and rate of its progression [18, 19], and is thus a biomechanical index for evaluating the efficacy of clinical interventions [8, 16, 17]. An increased KAM may lead to an increased compressive medial compartment load [20, 21], which is an important variable indicating the progress of medial knee OA. Apart from the changes in joint mechanics at the knee, peak hip flexor and abductor moments and ankle plantarflexor moments were also changed when compared to the control group [22]. Therefore, evaluating the efficacy of LW during gait should include changes in the biomechanical indices at the hip and ankle, which may also be found in persons with medial knee OA when a foot orthosis such as an LW is used [8, 15, 23].

The purpose of the current study was to investigate the immediate and long-term effects of LW insoles on the lower limb joint biomechanics, the KAM in particular, during gait in patients with bilateral medial knee OA. It was hypothesized that the LW insoles would immediately alter the loadings, not only at the knee, but also at the other joints during gait in persons with bilateral medial knee OA, and that long-term use of the LW insoles would change the joint biomechanics even during barefoot walking.

## Methods

### Subjects

Ten female subjects with bilateral medial knee OA (age 66 ± 5.3 years; height 156 ± 4.9 cm; mass 60 ± 5.1 kg; leg length 83.72 ± 3.64 cm; hip–knee–ankle angle 187.9° ± 2.5°) were recruited by an experienced physician (LFH) to participate in the current study. An a priori power analysis based on pilot results using GPOWER [24] determined that seven subjects would yield a power of 0.8 at a significance level of 0.05. All the subjects met the following inclusion criteria: (a) bilateral medial knee OA, (b) grade 2 or 3 in both knees according to the Kellgren/Lawrence (K/L) grading [25], and (c) ability to walk independently. A subject was excluded if she had (a) received treatment such as foot orthoses, intra-joint injections or an operation in the past 6 months, (b) other neuromusculoskeletal disorders, visual impairment or cognitive dysfunction that might disturb gait. The symptomatic severity of the subjects was evaluated using the Western Ontario and McMaster Universities Arthritis Index (WOMAC) [10], which comprises pain, stiffness and physical function sub-scales based on a 5-point Likert scale. All subjects gave informed written consent as approved by the Institutional Research Board.

### Laterally-wedged insoles

**Figure 1:**
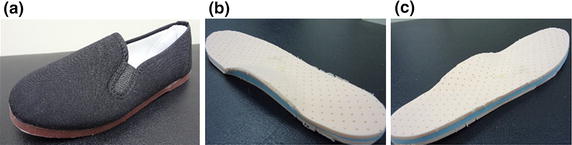
Experimental shoes and insoles used in the study. **a** The kung-fu shoes. **b** The laterally-wedged insoles (anterior-medial view). **c** The laterally-wedged insoles (posterior-lateral view).

The LW was made from ethylene vinyl acetate with a 7° lateral inclination (Figure 1b, c). The insoles were individually fabricated, with the lateral wedge extending along the entire length of the foot, and included the medial arch support. The arch support was located at a place underneath the foot determined using individual footprints and the height of the navicular tuberosity [8]. The original insoles in the personal walking shoes of each subject were removed and replaced by the corresponding LW insoles.

In the following 6 weeks after the LW insoles had been fitted, the subjects wore the LW insole whenever wearing shoes, for a period of 5–10 h each day [11]. The subjects received weekly telephone calls from the study investigators to determine whether there were any problems with adherence to the protocol or with discomfort with the shoes. Small adjustments to the shoes, such as a modification of the height and shape for the arch support, during the first week were permitted [26]. During the 6-week trial, participants were not discouraged from seeking additional treatments for OA, but were requested to record such changes in a daily log, including physical therapy and medication use, as well as any changes in the symptoms and any adverse effects from the insoles [27]. However, oral analgesia on the day of, or day before, a study visit was prohibited [6].

### Experimental protocol

The subjects were evaluated using gait analysis, both immediately after (Baseline) and 6 weeks after wearing the LW insoles (Follow-up). Each gait evaluation included assessment of the subjects under two conditions: barefoot condition and wearing kung-fu shoes (Figure 1a) with subject-specific LW insoles (LW condition). The subject was asked to walk at a self-selected pace on an 8-m walkway under barefoot and LW conditions in a random order determined by a random number table. Infrared retroreflective markers were attached on the anterior superior iliac spines, posterior superior iliac spines, greater trochanters, mid-thighs, lateral and medial femoral epicondyles, tibial tuberosities, fibular heads, lateral and medial malleoli, heels, navicular tuberosities and fifth metatarsal bases [28]. In the LW condition, small holes were made in the kung-fu shoes to place markers directly on the navicular tuberosities and fifth metatarsal bases. Several practice trials were allowed so that the subject could walk comfortably with the markers and LW along the walkway. During the experimental data collection, three-dimensional marker trajectories and GRF were captured by a 6-camera motion analysis system (Vicon MX 13, Oxford Group, UK) with a sampling rate of 120 Hz, and two forceplates (OR-6-7-1000, AMTI, USA) with a sampling rate of 1,080 Hz. Six successful trials, defined as those trials during which the subject did not step outside the forceplates, were collected for each test condition.

### Data analysis

For dynamic analysis, the pelvis-leg apparatus was modeled as a seven-link system. Each link were embedded with an orthogonal coordinate system with the positive x-axis directed anteriorly, the positive y-axis superiorly, and the positive z-axis to the right, following the convention recommended by the International Society of Biomechanics [29]. A Cardanic rotation sequence (z–x–y) was used to describe the rotational movements of each joint [30]. With the measured GRF and kinematic data, intersegmental forces and internal moments at the joints of the lower limbs were calculated using inverse dynamics. Inertial properties for each body segment, namely mass, center of mass and moment of inertia, were obtained using Dempster’s coefficients [31]. All the calculated moments were normalized to body weight and leg length. The center of pressure (COP) position was calculated using forces and moments measured by the forceplates. The medial/lateral (M/L) positions of the COP were described relative to the line of progression during a gait cycle, a positive value being to the side of the contralateral limb [8]. The frontal GRF was also calculated as the resultant force vector of the vertical and M/L component of the GRF, while the corresponding lever arm available at the knee was calculated as the perpendicular distance between the frontal GRF and the knee joint center. Gait speed and the foot progression angle, defined as the angle between the direction of progression and the long axis of the foot at midstance, were also obtained.

The peaks of the abductor moment at the knee during early and late stance phases (first and second peaks at P1 and P2), and the corresponding joint angles and moments at the hip, knee and ankle were extracted for subsequent statistical analysis. The magnitude of the frontal GRF and its lever arm available at the knee, and the M/L COP positions when the first and second peak KAM occurred, were also extracted.

### Statistical analysis

All the variables obtained for statistical analysis were tested for normality using the Shapiro–Wilk test. A paired *t* test was used to test the immediate effect of LW insoles (condition: barefoot vs. LW) on all the calculated biomechanical variables and to test the effect of 6-week use of LW insoles (evaluation time: Baseline vs. Follow-up) in terms of WOMAC scores and all the calculated biomechanical variables. All statistical analyses were performed using SPSS (version 19, Inc., Chicago, USA). All significance levels were set at α = 0.05.

## Results

The daily logs of the subjects revealed that none of them received any additional treatment for their OA knees and that they wore the LW insole for at least 6.5 h per day during the 6-week period. Significantly lower WOMAC scores were found after wearing the LW insoles for 6 weeks (pain: Baseline = 6.17 ± 2.76, Follow-up = 3.08 ± 2.07, *p* = 0.004, and physical function: Baseline = 20.47 ± 8.64, Follow-up = 10.14 ± 6.65, *p* = 0.013) while scores in joint stiffness remain unchanged (Baseline = 2.92 ± 1.68, Follow-up = 2.67 ± 1.61; *p* = 0.343).

Temporal–spatial parameters, namely stride duration, walking speed, step width and step length, were not significantly different between barefoot and LW conditions, either at Baseline or Follow-up (Table 1). The foot progression angles between barefoot and LW conditions were not significantly different between Baseline and Follow-up. Compared with Baseline, foot progression angles in the barefoot condition were significantly decreased at Follow-up but those during the LW condition were not statistically changed (Table 1).

**Table 1:** Temporal–spatial parameters

	Conditions	Evaluation time				*p* value		
		Baseline		Follow-up		Barefoot vs. LW at Baseline	Barefoot vs. LW at Follow-up	Baseline vs. Follow-up
		Mean	SD	Mean	SD			
Walking time (s)	Barefoot	1.95	0.19	1.66	0.10	0.81	0.18	0.06
	LW	1.98	0.27	1.85	0.31			0.46
Walking speed (m/s)	Barefoot	0.77	0.10	0.90	0.11	0.91	0.41	0.08
	LW	0.78	0.12	0.85	0.12			0.25
Step length (%LL)	Barefoot	60.42	2.54	61.79	3.37	0.80	0.28	0.46
	LW	60.06	2.35	69.23	15.84			0.12
Step width (%LL)	Barefoot	10.53	3.33	13.60	3.02	0.52	0.40	0.12
	LW	11.83	3.47	12.00	3.29			0.60
Foot progression angle (°)	Barefoot	5.44	1.77	3.61	1.97	0.67	0.20	0.04*
	LW	4.92	2.42	5.66	3.11			0.35

*SD* standard deviation, *LW* laterally-wedged, *LL* leg length.

* Significant difference Baseline versus Follow-up during barefoot condition.

The angles and moments at P1 and P2 at the hip for the barefoot and LW conditions were not significantly different, neither at Baseline, nor at Follow-up (Table 2). When compared to those at Baseline, in both the barefoot and LW conditions, the hip angles and moments at P1 and P2 were not statistically changed at Follow-up (Table 2), while during the barefoot condition the external rotation of the hip at heelstrike was significantly lower at Follow-up compared with that at Baseline (Baseline = 10.33 ± 5.11, Follow-up = 3.29 ± 3.91, p = 0.017).

**Table 2:** Joint angles and moments at the hip

	Conditions	Evaluation time				*p* value		
		Baseline		Follow-up		Barefoot vs. LW at Baseline	Barefoot vs. LW at Follow-up	Baseline vs. Follow-up
		Mean	SD	Mean	SD			
First peak KAM								
Hip adduction (+)/abduction (−) angle (°)	Barefoot	7.57	2.67	7.61	2.82	0.66	0.32	0.91
	LW	7.01	1.54	5.91	2.88			0.46
Hip internal rotation (+)/external rotation (−) angle (°)	Barefoot	−1.28	4.92	−2.11	2.71	0.39	0.48	0.75
	LW	0.91	3.37	−1.04	2.35			0.46
Hip flexion (+)/extension (−) angle (°)	Barefoot	9.78	4.55	13.56	4.43	0.66	0.28	0.35
	LW	10.75	2.70	10.75	4.27			0.91
Hip adductor (+)/abductor (−) moment (%BW*LL)	Barefoot	11.44	1.14	10.53	3.24	0.80	0.51	0.91
	LW	11.29	0.80	11.55	1.73			0.60
Hip external rotator (+)/internal rotator (−) moment (%BW*LL)	Barefoot	0.37	0.57	0.73	0.86	0.79	0.67	0.46
	LW	0.29	0.42	0.54	0.63			0.75
Hip extensor (+)/flexor (−) moment (%BW*LL)	Barefoot	1.63	1.06	0.54	2.02	0.48	0.54	0.11
	LW	0.95	2.04	1.57	3.50			0.92
Second peak KAM								
Hip adduction (+)/abduction (−) angle (°)	Barefoot	5.52	2.30	4.63	2.40	0.19	0.10	0.60
	LW	3.78	2.00	2.47	1.76			0.06
Hip internal rotation (+)/external rotation (−) angle (°)	Barefoot	3.81	5.07	1.98	2.79	0.52	0.25	0.35
	LW	5.39	2.97	3.78	2.37			0.46
Hip flexion (+)/extension (−) angle (°)	Barefoot	−5.08	1.70	0.02	6.07	0.17	0.10	0.09
	LW	−2.86	3.32	−5.50	4.36			0.12
Hip adductor (+)/abductor (−) moment (%BW*LL)	Barefoot	10.86	0.76	8.59	2.78	0.21	0.32	0.12
	LW	10.03	1.34	10.00	1.86			0.75
Hip external rotator (+)/internal rotator (−) moment (%BW*LL)	Barefoot	−1.52	0.70	−0.94	1.16	0.95	0.33	0.25
	LW	−1.50	0.48	−1.53	0.83			0.92
Hip extensor (+)/flexor (−) moment (%BW*LL)	Barefoot	−1.97	1.79	−2.49	3.05	0.94	0.82	0.46
	LW	−2.04	2.06	−1.99	4.27			0.92

*SD* standard deviation, *LW* laterally-wedged, *LL* leg length, *BW* body weight.

**Figure 2:**
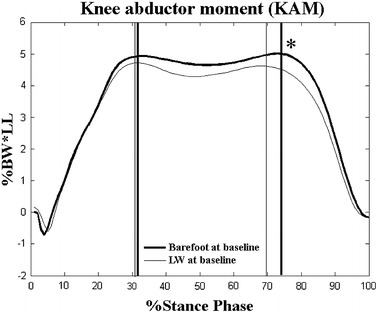
Effect of LW on peak knee abductor moments (KAM) at Baseline. Ensemble-averaged patterns of knee abductor moments during the barefoot condition (*thick lines*) and during the laterally-wedged condition (LW, *thin lines*) at Baseline. *Vertical lines* indicate the instances in time when the first and second peaks KAM occurred; %BW*LL: percentage of leg length multiplied by body weight; *significant LW effects *p* < 0.05.

**Figure 3:**
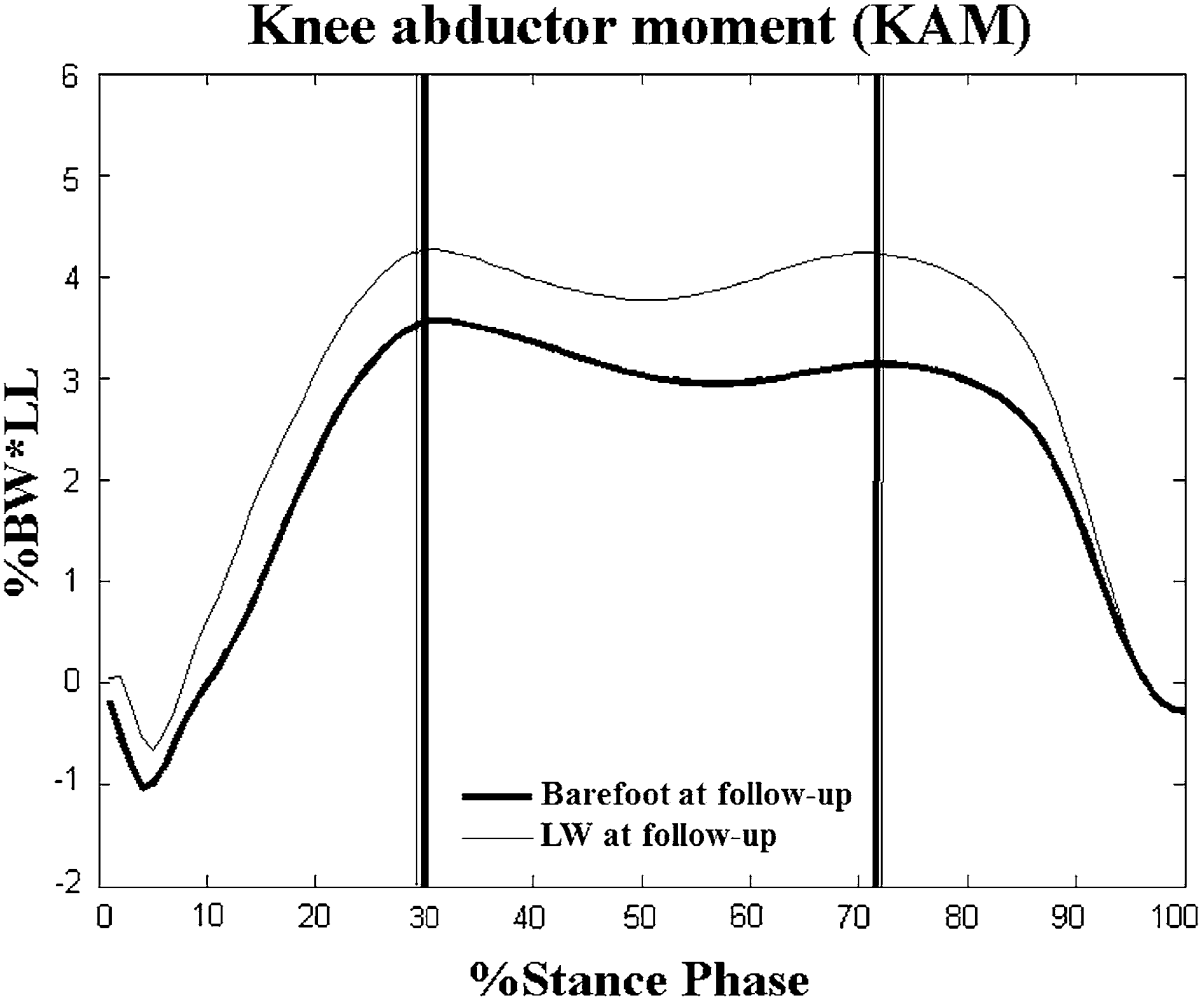
Effect of LW on peak knee abductor moments (KAM) at Follow-up. Ensemble-averaged patterns of knee abductor moments during the barefoot condition (*thick lines*) and during the laterally-wedged condition (LW, *thin lines*) at Follow-up. *Vertical lines* indicate the instances in time when the first and second peaks KAM occurred; %BW*LL: percentage of leg length multiplied by body weight.

**Figure 4:**
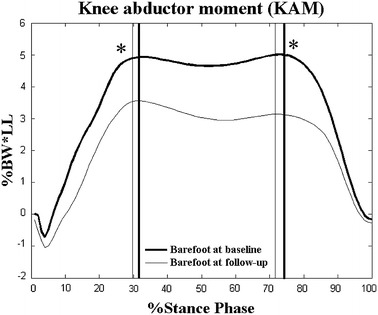
Long-term effect on peak knee abductor moments (KAM) during the barefoot condition. Ensemble-averaged patterns of knee abductor moments at Baseline (*thick lines*) and at Follow-up (LW, *thin lines*) during the barefoot condition. *Vertical lines* indicate the instances in time when the first and second peaks KAM occurred; %BW*LL: percentage of leg length multiplied by body weight; *significant long-term effects *p* < 0.05.

**Figure 5:**
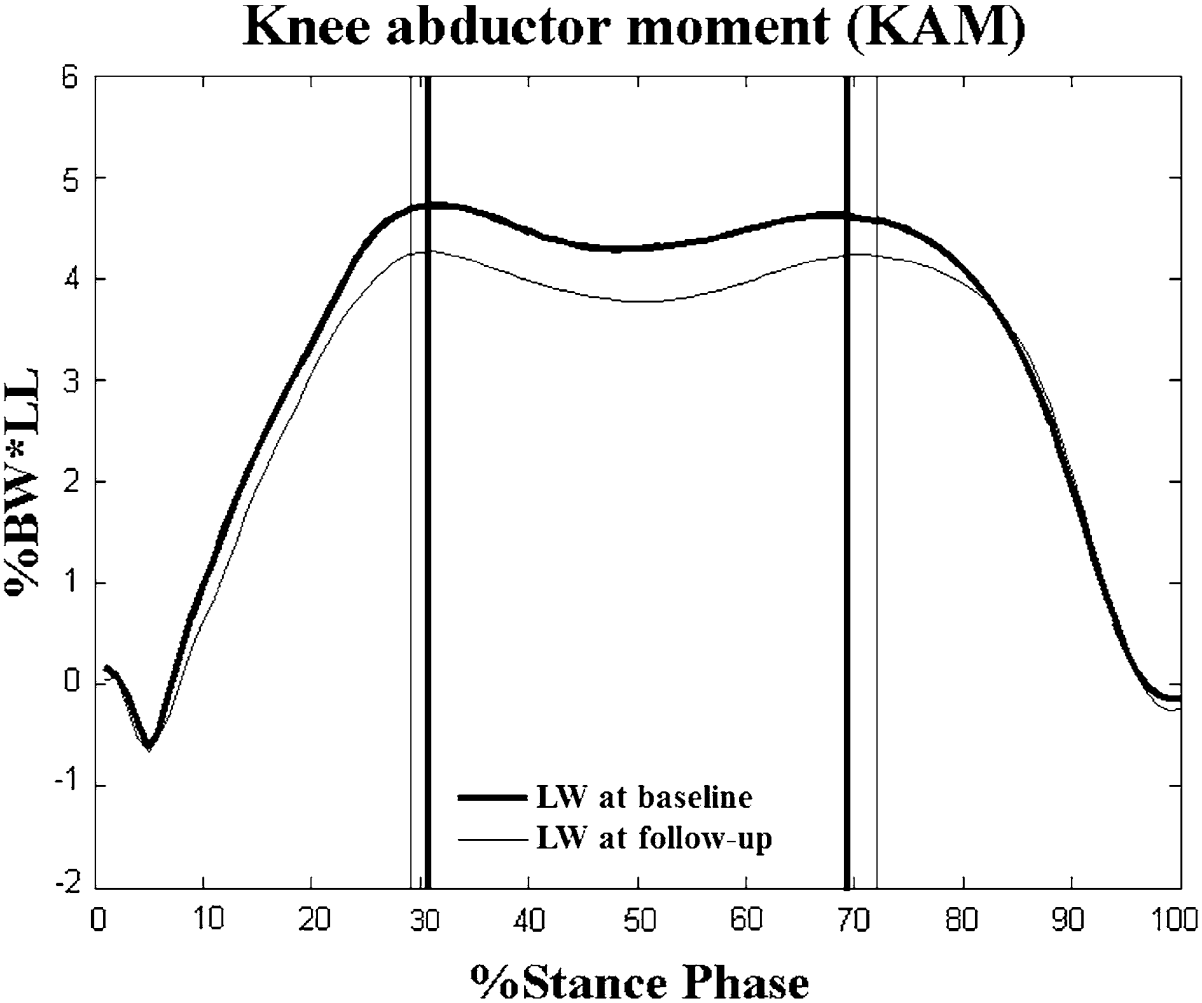
Long-term effect on peak knee abductor moments (KAM) during the laterally-wedged condition. Ensemble-averaged patterns of knee abductor moments at Baseline (*thick lines*) and at Follow-up (LW, *thin lines*) during the laterally-wedged condition. Vertical lines indicate the instances in time when the first and second peaks KAM occurred; %BW*LL: percentage of leg length multiplied by body weight.

The angles and moments at P1 and P2 at the knee for the barefoot and LW conditions were not significantly different, neither at Baseline, nor at Follow-up (Tables 3). The second peaks of KAM during the LW conditions were significantly lower than those during the barefoot conditions at Baseline (*p* < 0.05, Table 3; Figure 2), while the first and second peaks of KAM between barefoot and LW conditions were not significantly different at Follow-up (*p* > 0.05, Table 3; Figure 3). On the other hand, the flexion angle at the knee at P1 and P2 in the barefoot condition was significantly increased at Follow-up when compared to Baseline (*p* < 0.05, Table 3). The two peaks in the barefoot condition were significantly decreased at Follow-up (*p* < 0.05, Table 3; Figure 4) but not statistically changed in the LW condition when compared to those at Baseline (*p* > 0.05, Table 3; Figure 5).

**Table 3:** Joint angles and moments at the knee

	Conditions	Evaluation time				*p* value		
		Baseline		Follow-up		Barefoot vs. LW at Baseline	Barefoot vs. LW at Follow-up	Baseline vs. Follow-up
		Mean	SD	Mean	SD			
First peak KAM								
Knee adduction (+)/abduction (−) angle (°)	Barefoot	0.72	1.70	−1.48	3.90	0.51	0.79	0.25
	LW	0.01	1.93	−0.92	3.42			0.25
Knee internal rotation (+)/external rotation (−) angle (°)	Barefoot	2.23	1.57	1.62	4.20	0.53	0.40	0.91
	LW	2.85	1.77	3.79	4.47			0.75
Knee flexion (+)/extension (−) angle (°)	Barefoot	9.41	4.31	16.58	4.90	0.37	0.19	0.03^†^
	LW	11.91	4.90	13.26	3.32			0.25
Knee abductor (+)/adductor (−) moment (%BW*LL)	Barefoot	5.14	0.71	3.69	1.55	0.15	0.35	0.03^†^
	LW	4.71	0.67	4.21	0.96			0.25
Knee external rotator (+)/internal rotator (−) moment (%BW*LL)	Barefoot	−0.70	0.33	−0.54	0.21	0.62	0.85	0.35
	LW	−0.60	0.33	−0.52	0.25			0.46
Knee extensor (+)/flexor (−) moment (%BW*LL)	Barefoot	1.57	0.96	3.15	2.48	0.46	0.89	0.17
	LW	2.24	1.91	2.97	1.84			0.25
Second peak KAM								
Knee adduction (+)/abduction (−) angle (°)	Barefoot	1.22	1.49	−1.15	3.86	0.7	0.57	0.17
	LW	0.85	1.87	0.00	2.91			0.17
Knee internal rotation (+)/external rotation (−) angle (°)	Barefoot	3.64	2.53	3.48	3.98	0.78	0.32	0.60
	LW	4.05	2.56	5.59	3.10			0.60
Knee flexion (+)/extension(−) angle (°)	Barefoot	7.53	2.89	13.24	5.28	0.44	0.16	0.04^†^
	LW	9.36	4.83	9.26	3.92			0.75
Knee abductor (+)/adductor (−) moment (%BW*LL)	Barefoot	5.16	0.71	3.26	1.76	0.04*	0.23	0.02^†^
	LW	4.53	0.67	4.04	0.60			0.25
Knee external rotator (+)/internal rotator (−) moment (%BW*LL)	Barefoot	−1.83	0.36	−1.25	0.63	0.31	0.28	0.12
	LW	−1.61	0.37	−1.54	0.15			0.75
Knee extensor(+)/flexor (−) moment (%BW*LL)	Barefoot	−1.63	1.09	−0.54	1.94	0.94	0.21	0.46
	LW	−1.67	0.89	−1.62	0.49			0.92

*SD* standard deviation, *LW* laterally-wedged, *LL* leg length, *BW* body weight.

* Significant difference barefoot versus LW at Baseline.

^†^Significant difference Baseline versus Follow-up during barefoot condition.

The angles and moments at P1 and P2 at the ankle for the barefoot and LW conditions were not significantly different, neither at Baseline, nor at Follow-up (Table 4); When compared to those at Baseline, in the barefoot conditions, the ankle dorsiflexion angles at P1 was significantly increased at Follow-up (*p* < 0.05, Table 4). When compared to those at Baseline, in the LW condition, the ankle eversion at P1 and invertor moments at P2 were significantly increased at Follow-up (*p* < 0.05, Table 4).

**Table 4:** Joint angles and moments at the ankle

	Conditions	Evaluation time				*p* value		
		Baseline		Follow-up		Barefoot vs. LW at Baseline	Barefoot vs. LW at Follow-up	Baseline vs. Follow-up
		Mean	SD	Mean	SD			
First peak KAM								
Ankle inversion(+)/eversion (−) angle (°)	Barefoot	−1.47	3.20	−4.19	3.02	0.36	0.42	0.25
	LW	−0.06	1.74	−3.12	0.78			0.03^†^
Ankle internal rotation (+)/external rotation (−) angle (°)	Barefoot	1.03	1.08	1.63	1.46	0.96	0.21	0.25
	LW	1.01	1.17	0.77	0.69			0.60
Ankle dorsiflexion (+)/plantarflexion (−) angle (°)	Barefoot	1.64	1.15	4.66	1.09	0.11	0.16	0.03*
	LW	0.24	1.63	3.43	1.67			0.05
Ankle evertor (+)/invertor(−) moment (%BW*LL)	Barefoot	−0.27	0.28	−0.59	0.48	0.42	0.83	0.35
	LW	−0.39	0.19	−0.55	0.28			0.05
Ankle external rotator (+)/internal rotator (−) moment (%BW*LL)	Barefoot	−1.64	0.82	−1.27	0.64	0.69	0.97	0.46
	LW	−1.45	0.81	−1.26	0.72			0.75
Ankle plantarflexor (+)/dorsiflexor (−) moment (%BW*LL)	Barefoot	4.82	1.74	4.49	1.83	0.46	0.96	0.75
	LW	4.01	1.98	4.54	2.09			0.12
Second peak KAM								
Ankle inversion(+)/eversion (−) angle (°)	Barefoot	−0.87	3.21	−3.65	3.37	0.96	0.42	0.25
	LW	−0.80	2.21	−2.44	1.14			0.12
Ankle internal rotation (+)/external rotation (−) angle (°)	Barefoot	3.15	0.87	3.57	1.60	0.76	0.74	0.46
	LW	2.94	1.40	3.28	1.36			0.60
Ankle dorsiflexion (+)/plantarflexion (−) angle (°)	Barefoot	6.41	2.22	8.59	1.64	0.78	0.28	0.17
	LW	6.06	2.04	7.52	1.65			0.05
Ankle evertor(+)/invertor (−) moment (%BW*LL)	Barefoot	−0.53	0.47	−0.93	0.85	0.78	0.55	0.46
	LW	−0.60	0.37	−0.68	0.50			0.04^†^
Ankle external rotator (+)/internal rotator (−) moment (%BW*LL)	Barefoot	−4.23	1.44	−3.22	1.27	0.75	0.43	0.17
	LW	−3.98	1.50	−3.86	1.44			0.60
Ankle plantarflexor (+)/dorsiflexor (−) moment (%BW*LL)	Barefoot	12.23	1.59	11.15	3.49	0.19	0.43	0.60
	LW	11.16	1.01	12.51	2.26			0.35

*SD* standard deviation, *LW* laterally-wedged, *LL* leg length, *BW* body weight.

* Significant difference Baseline versus Follow-up during barefoot condition.

^†^Significant difference Baseline versus Follow-up during LW condition.

The magnitudes of the frontal GRF at P1 and P2 between barefoot and LW conditions were not significantly different at Baseline and at Follow-up (*p* > 0.05, Table 5). The magnitudes of the frontal GRF at P1 and P2 in both the barefoot and LW conditions at Follow-up were not statistically changed when compared to those at Baseline (*p* > 0.05, Table 5). The lever arm lengths of the frontal GRF at P1 and P2 during the LW condition were significantly lower than those during the barefoot condition at Baseline (*p* < 0.05, Table 5). The lever arm lengths of the frontal GRF at P1 and P2 between barefoot and LW conditions were not significantly different at Follow-up (*p* > 0.05, Table 5). The lever arm lengths of the frontal GRF at P1 and P2 for the barefoot condition were significantly decreased at Follow-up (*p* < 0.05, Table 5) but those in the LW condition were not statistically changed when compared to the Baseline (*p* > 0.05, Table 5).

**Table 5:** Frontal ground reaction force (GRF) and lever arm

	Conditions	Evaluation time				*p* value		
		Baseline		Follow-up		Barefoot vs. LW at Baseline	Barefoot vs. LW at Follow-up	Baseline vs. Follow-up
		Mean	SD	Mean	SD			
First peak KAM								
Frontal GRF (%BW)	Barefoot	93.86	6.39	90.98	11.42	0.18	0.79	0.91
	LW	97.94	2.86	88.81	15.76			0.25
Frontal lever arm (%LL)	Barefoot	4.59	1.15	3.26	1.01	0.03*	0.61	0.02^†^
	LW	2.00	1.14	2.15	1.44			0.60
Second peak KAM								
Frontal GRF (%BW)	Barefoot	98.78	3.40	90.78	12.59	1.00	0.93	0.25
	LW	98.78	1.71	90.16	16.34			0.75
Frontal lever arm (%LL)	Barefoot	4.17	1.28	3.09	1.44	0.03*	0.56	0.02^†^
	LW	2.67	1.29	2.78	1.51			0.92

*SD* standard deviation, *LW* laterally-wedged, *LL* leg length, *BW* body weight, *GRF* ground reaction force

* Significant difference barefoot versus LW at Baseline.

^†^Significant difference Baseline versus Follow-up during barefoot condition.

The COP positions at P1 and P2 in the LW condition were laterally shifted significantly more than those in the barefoot condition, both at Baseline and at Follow-up (*p* < 0.05, Table 6). However, the COP positions in the barefoot and LW conditions were not statistically changed at Follow-up when compared to Baseline (*p* > 0.05, Table 6).

**Table 6:** Medial–lateral (M/L) center of pressure (COP) position

	Conditions	Evaluation time				*p* value		
		Baseline		Follow-up		Barefoot vs. LW at Baseline	Barefoot vs. LW at Follow-up	Baseline vs. Follow-up
		Mean	SD	Mean	SD			
First peak KAM								
M(+)/L(−) COP position (%LL)	Barefoot	−1.86	0.31	−1.84	0.52	0.00*	0.02^†^	0.91
	LW	−2.93	0.44	−2.71	0.44			0.46
Second peak KAM								
M(+)/L(−) COP position (%LL)	Barefoot	−1.45	0.53	−1.47	0.71	0.00*	0.18	0.66
	LW	−2.36	0.40	−2.10	0.80			0.46

*SD* standard deviation, *LW* laterally-wedged, *LL* leg length, *COP* center of pressure.

* Significant difference barefoot versus LW at Baseline.

^†^Significant difference barefoot versus LW at Follow-up.

## Discussion

This study aimed to evaluate the immediate and long-term efficacy of the LW insoles during gait in persons with bilateral medial knee OA during both LW and barefoot conditions. The results showed that, after prolonged use of LW insoles, a specific gait adaptation with reduced knee loading was found when not wearing LW insoles. Significantly lower WOMAC scores were also found, both in pain and physical function scores, suggesting that subjects with knee OA responded favorably to LW insoles. These results agree with a previous one-year prospective controlled trial by Barrios et al. [6]. The simultaneous improvement in both pain and physical function may be explained in part by the fact that pain relief enhances physical function [9].

The significantly reduced second peak KAM when wearing LW insoles compared to the barefoot condition at Baseline suggests that the LW insoles were effective in reducing unfavorable loadings at the knee immediately upon wearing LW insoles (Figure 2; Table 3). The immediate effects observed in the current study were mainly as a result of the lateral shift of the COP (Table 6), combined with a reduced frontal plane GRF lever arm after wearing the LW insoles (Table 5). Reduced peak KAM is helpful for pain relief [32] in persons with knee OA who have greater peak KAM than normal controls [8, 33–35]. The current results were consistent with previous studies in patients with knee OA [8] and healthy adults [8, 17], although different from a study that reported no immediate efficacy on the reduction of KAM [15]. These controversies may be related to multiple factors, including the symmetry of the alignment of the knees in the patients studied. In the study by Abdallah and Radwan [15], they compared the results between unilateral and bilateral use of the LW insoles. They included patients with unilateral and bilateral knee OA with underlying asymmetrical knee alignment that could not be removed, neither with unilateral, nor bilateral use of LW insoles. The difference in patient selection may in part explain the differences in the findings. In the current study the asymmetrical issue was minimized as only patients with bilateral knee OA were recruited. Therefore, the current findings were considered to be a plausible support for the immediate efficacy of LW insoles. Nonetheless, the resolution of the controversies of the immediate effects of LW may not be of great clinical significance because the insoles are often prescribed for long-term use. Thus, the evaluation of the long-term efficacy of LW insoles in gait improvement seems more important for clinical applications.

After 6 weeks of wearing LW insoles, no significant changes were found in most of the biomechanical parameters—including KAM—during the LW condition when compared to Baseline, suggesting that the reduction of KAM with LW insoles found at Baseline was maintained throughout this period. The trade-off for maintaining these positive long-term effects was slightly increased eversion angles and invertor moments at the ankle. Previous studies also found that the reduction of the harmful KAM immediately after wearing LW insoles was accompanied with increased ankle invertor moments [15, 36], and both these positive and harmful effects were maintained after a prolonged, continuous use of LW insoles [12], These increased ankle invertor moments after prolonged use of the LW orthoses suggest an increased demand on the relevant muscles, which may lead to early fatigue and/or over-use injuries. Therefore, strengthening the ankle invertor muscles and monitoring their condition may be necessary to reduce any potential discomfort of the foot and any adverse effects if the LW insoles are used long-term for persons with bilateral medial knee OA.

While the harmful KAM was not further reduced after 6 weeks of wearing the LW insoles, a specific gait adaptation with reduced knee loading was revealed when walking without LW insoles, i.e., barefoot (Figure 4; Table 3). This suggests that the LW insoles also served as a device for developing a gait pattern that will reduce the KAM. In other words, in the 6-week period the patient acquired a specific gait pattern to reduce the KAM without having to rely on wearing the LW insoles. This was achieved by adopting a strategy that involved reduced external rotation of the hip at heelstrike and increased ankle eversion during stance, which led to significantly reduced lever arm lengths of the frontal GRF available at the knee and thus a lesser KAM. A previous study has shown that an increased foot progression angle, presumably as a result of increased external hip rotation at heelstrike was the strategy used by the knee OA patients to reduce KAM immediately upon wearing the LW insoles [8]. After prolonged use of the LW insoles, the current patients reduced the external rotation angle of the hip at heelstrike with slightly increased ankle eversion during stance, suggesting that they had changed from the previously observed hip strategy to one that relies more on the ankle. This shift of compensatory changes from the hip towards the ankle indicates a learning process for a more localized strategy. This acquired localized strategy, however, may increase the chances of potential discomfort of the foot and may have an adverse effect on the ankle. To avoid early fatigue and possible over-use injuries when walking with the adapted gait patterns after prolonged use of LW insoles, it is suggested that monitoring the foot contact patterns and the condition of the ankle invertor muscles should be included in the rehabilitation program in persons with bilateral medial knee OA. Meanwhile, previous studies showed that patients with knee OA walked with reduced knee flexion [2, 37] so the significantly increased knee flexion in the barefoot condition at Follow-up when compared to Baseline indicated another improvement of gait patterns after long-term use of LW insoles.

The durations of LW usage in previous studies on the long-term effects of LW insoles ranged from 2 weeks to 2 years. The current findings showed that significant changes in knee loading could be observed after 6 weeks of wearing LW insoles. Longer period may be used but the effects of the insoles wearing out would then have to be taken into account. The observed gait adaptation indicated for the first time in the literature the learning effects of using LW insoles. Further study is needed to determine for how long the acquired gait patterns could be maintained if the use of the LW insoles were discontinued.

Since multiple *t* tests were performed in the current study, the possibility of significance level inflation and type 1 error should be noted. While methods such as Bonferroni corrections could be used to deal with this issue, the likelihood of type II errors is also increased so that truly important differences are deemed non-significant [38]. Therefore, instead of correcting the significance level, we described the tests of significance performed, reported the p-values, and discussed the possible interpretations of each result, following suggestions in the literature [38].

## Conclusions

Immediate and long-term effects of LW insoles on the abductor moments at the knee and the compensatory changes at the other joints in persons with bilateral medial knee OA during walking were studied. The learning effects on gait patterns from long-term wearing of LW insoles were quantified for the first time in the literature using computerized gait analysis techniques. After long-term LW insole use, the pain and physical function were improved as a result of decreased peak KAM, indicating a positive long-term effect in persons with bilateral medial knee OA. In addition to being an orthosis, the LW insoles also serve as a treatment intervention that may alter gait patterns in favor of reduced KAM. The adverse effects associated the increase of ankle eversion angles and invertor moments suggest the necessity of strengthening the ankle invertor muscles and monitoring their condition when the LW is used as a long-term intervention for persons with bilateral medial knee OA.

## Abbreviations

OA: osteoarthritis
LW: laterally-wedged
GRF: ground reaction force
KAM: knee abductor moment
WOMAC: Western Ontario and McMaster Universities Arthritis Index
COP: center of pressure.

## References

[1] Toda Y, Tsukimura N, Segal N (2005). An optimal duration of daily wear for an insole with subtalar strapping in patients with varus deformity osteoarthritis of the knee. Osteoarthr Cartil.

[2] Wei IP, Hsu WC, Chien HL, Chang CF, Liu YH, Ho TJ (2009). Leg and joint stiffness in patients with bilateral medial knee osteoarthritis during level walking. J Mech.

[3] Wang TM, Hsu WC, Chang CF, Hu CC, Lu TW (2010). Effects of knee osteoarthritis on body’s center of mass motion in older adults during level walking. Biomed Eng-Appl Basis Commun.

[4] Hsu WC, Wang TM, Liu MW, Chang CF, Chen HL, Lu TW (2010). Control of body’s center of mass motion during level walking and obstacle-crossing in older patients with knee osteoarthritis. J Mech.

[5] Yildiz N, Topuz O, Gungen GO, Deniz S, Alkan H, Ardic F (2010). Health-related quality of life (Nottingham Health Profile) in knee osteoarthritis: correlation with clinical variables and self-reported disability. Rheumatol Int.

[6] Barrios JA, Crenshaw JR, Royer TD, Davis IS (2009). Walking shoes and laterally wedged orthoses in the clinical management of medial tibiofemoral osteoarthritis: a one-year prospective controlled trial. Knee.

[7] van Raaij TM, Reijman M, Brouwer RW, Bierma-Zeinstra SM, Verhaar JA (2010). Medial knee osteoarthritis treated by insoles or braces: a randomized trial. Clin Orthop Relat Res.

[8] Yeh HC, Chen LF, Hsu WC, Lu TW, Hsieh LF, Chen HL (2014). Immediate efficacy of laterally wedged insoles with arch support on walking in persons with bilateral medial knee osteoarthritis. Arch Phys Med Rehabil.

[9] Baker K, Goggins J, Xie H, Szumowski K, LaValley M, Hunter DJ (2007). A randomized crossover trial of a wedged insole for treatment of knee osteoarthritis. Arthritis Rheum.

[10] Pham T, Maillefert JF, Hudry C, Kieffert P, Bourgeois P, Lechevalier D (2004). Laterally elevated wedged insoles in the treatment of medial knee osteoarthritis. A two-year prospective randomized controlled study. Osteoarthr Cartil.

[11] Toda Y, Tsukimura N (2006). A 2-year follow-up of a study to compare the efficacy of lateral wedged insoles with subtalar strapping and in-shoe lateral wedged insoles in patients with varus deformity osteoarthritis of the knee. Osteoarthr Cartil.

[12] Hinman RS, Bowles KA, Bennell KL (2009). Laterally wedged insoles in knee osteoarthritis: do biomechanical effects decline after one month of wear?. BMC Musculoskelet Disord.

[13] Hinman RS, Payne C, Metcalf BR, Wrigley TV, Bennell KL (2008). Lateral wedges in knee osteoarthritis: what are their immediate clinical and biomechanical effects and can these predict a three-month clinical outcome?. Arthritis Care Res.

[14] Segal NA, Foster NA, Dhamani S, Ohashi K, Yack HJ (2009). Effects of concurrent use of an ankle support with a laterally wedged insole for medial knee osteoarthritis. PM&R.

[15] Abdallah AA, Radwan AY (2011). Biomechanical changes accompanying unilateral and bilateral use of laterally wedged insoles with medial arch supports in patients with medial knee osteoarthritis. Clin Biomech.

[16] Jones RK, Zhang M, Laxton P, Findlow AH, Liu A (2013). The biomechanical effects of a new design of lateral wedge insole on the knee and ankle during walking. Hum Mov Sci.

[17] Nakajima K, Kakihana W, Nakagawa T, Mitomi H, Hikita A, Suzuki R (2009). Addition of an arch support improves the biomechanical effect of a laterally wedged insole. Gait Posture.

[18] Miyazaki T, Wada M, Kawahara H, Sato M, Baba H, Shimada S (2002). Dynamic load at baseline can predict radiographic disease progression in medial compartment knee osteoarthritis. Ann Rheum Dis.

[19] Mundermann A, Dyrby CO, Hurwitz DE, Sharma L, Andriacchi TP (2004). Potential strategies to reduce medial compartment loading in patients with knee osteoarthritis of varying severity: reduced walking speed. Arthritis Rheum.

[20] Erhart JC, Dyrby CO, D’Lima DD, Colwell CW, Andriacchi TP (2010). Changes in in vivo knee loading with a variable-stiffness intervention shoe correlate with changes in the knee adduction moment. J Orthop Res.

[21] Zhao D, Banks SA, Mitchell KH, D’Lima DD, Colwell CW, Fregly BJ (2007). Correlation between the knee adduction torque and medial contact force for a variety of gait patterns. J Orthop Res.

[22] Jenkyn TR, Hunt MA, Jones IC, Giffin JR, Birmingham TB (2008). Toe-out gait in patients with knee osteoarthritis partially transforms external knee adduction moment into flexion moment during early stance phase of gait: a tri-planar kinetic mechanism. J Biomech.

[23] Butler RJ, Barrios JA, Royer T, Davis IS (2009). Effect of laterally wedged foot orthoses on rearfoot and hip mechanics in patients with medial knee osteoarthritis. Prosthet Orthot Int.

[24] Erdfelder E, Faul F, Buchner A (1996). GPOWER: a general power analysis program. Behav Res Meth Instr.

[25] Kellgren JH, Lawrence JS (1957). Radiological assessment of osteo-arthrosis. Ann Rheum Dis.

[26] Shakoor N, Lidtke RH, Wimmer MA, Mikolaitis RA, Foucher KC, Thorp LE (2013). Improvement in knee loading after use of specialized footwear for knee osteoarthritis: results of a six-month pilot investigation. Arthritis Rheum.

[27] Turpin KM, De Vincenzo A, Apps AM, Cooney T, MacKenzie MD, Chang R (2012). Biomechanical and clinical outcomes with shock-absorbing insoles in patients with knee osteoarthritis: immediate effects and changes after 1 month of wear. Arch Phys Med Rehabil.

[28] Lu TW, Wei IP, Liu YH, Hsu WC, Wang TM, Chang CF, Lin JG (2010). Immediate effects of acupuncture on gait patterns in patients with knee osteoarthritis. Chin Med J (Engl).

[29] Wu G, Cavanagh PR (1995). ISB recommendations for standardization in the reporting of kinematic data. J Biomech.

[30] Cole GK, Nigg BM, Ronsky JL, Yeadon MR (1993). Application of the joint coordinate system to three-dimensional joint attitude and movement representation: a standardization proposal. J Biomech Eng.

[31] Dempster WT, Gabel WC, Felts WJ (1959). The anthropometry of the manual work space for the seated subject. Am J Phys Anthropol.

[32] Erhart-Hledik JC, Elspas B, Giori NJ, Andriacchi TP (2012). Effect of variable-stiffness walking shoes on knee adduction moment, pain, and function in subjects with medial compartment knee osteoarthritis after 1 year. J Orthop Res.

[33] Baliunas AJ, Hurwitz DE, Ryals AB, Karrar A, Case JP, Block JA (2002). Increased knee joint loads during walking are present in subjects with knee osteoarthritis. Osteoarthr Cartil.

[34] Gok H, Ergin S, Yavuzer G (2002). Kinetic and kinematic characteristic of gait in patients with medial knee arthrosis. Acta Orthop Scand.

[35] Kaufman KR, Hughes C, Morrey BF, Morrey M, An KN (2001). Gait characteristics of patients with knee osteosrthritis. J Biomech.

[36] Kakihana W, Akai M, Nakazawa K, Takashima T, Naito K, Torii S (2005). Effects of laterally wedged insoles on knee and subtalar joint moments. Arch Phys Med Rehabil.

[37] Huang SC, Wei IP, Chien HL, Wang TM, Liu YH, Chen HL (2008). Effects of severity of degeneration on gait patterns in patients with medial knee osteoarthritis. Med Eng Phys.

[38] Perneger TV (1998). What’s wrong with Bonferroni adjustments. BMJ.

